# Effects of dietary level of tannic acid and protein on internal organ weights and biochemical blood parameters of rats

**DOI:** 10.1371/journal.pone.0190769

**Published:** 2018-01-05

**Authors:** Marcin Barszcz, Marcin Taciak, Anna Tuśnio, Jacek Skomiał

**Affiliations:** Department of Animal Nutrition, The Kielanowski Institute of Animal Physiology and Nutrition, Polish Academy of Sciences, Jabłonna, Poland; Leibniz-Institut fur Pflanzengenetik und Kulturpflanzenforschung Gatersleben, GERMANY

## Abstract

Tannic acid (TA) is a polyphenolic compound with a health-promoting potential for humans. It is hypothesised that TA effects on the relative weight of internal organs and biochemical blood indices are modified by dietary protein level in rats. The study involved 72 rats divided into 12 groups fed diets with 10 or 18% of crude protein (CP) and supplemented with 0, 0.25, 0.5, 1, 1.5 or 2% of TA. After 3 weeks of feeding, the relative weight of the caecum was greater in rats fed TA diets, while feeding diets with 10% of CP increased the relative weight of the stomach, small intestine and caecum, but decreased that of kidneys and spleen. Albumin concentration was higher in rats fed 0.25% and 0.5% TA diets than in rats given the 2% TA diets. The 2% TA diets reduced creatine kinase (CK) activity compared to non-supplemented diets and those with 0.5, 1 and 1.5% of TA. Rats fed the 10% CP diets had a higher activity of alkaline phosphatase, amylase, and γ-glutamyltransferase as well as the concentration of iron and cholesterol, but lower that of urea and uric acid. The interaction affected only cholinesterase activity. In conclusion, TA induced caecal hypertrophy and could act as a cardioprotective agent, as demonstrated by reduced CK activity, but these effects were not modified by dietary protein level.

## Introduction

Tannic acid (TA) is a polyphenolic compound consisting of a central glucose molecule, which hydroxyl groups are linked by ester bonds with gallic acid moieties. One molecule contains 8 to 10 moles of gallic acid per mole of glucose [1]. TA is a well-known representative of gallotannins, a class of hydrolysable tannins, which are widely distributed throughout the feeds and foods of plant origin [2]. TA is a potent antioxidant with antimutagenic and anticarcinogenic properties. Thus, it may protect DNA against oxidative damage and may be useful in the therapy of diseases related to oxidative stress such as colorectal cancer and heart and liver damage [3–5]. Its health-promoting potential may be applied in farm animal nutrition, as previously shown for gallotannin preparation that improved feed efficiency and reduced proteolytic fermentation in the caecum of piglets [6]. However, TA forms insoluble complexes with dietary protein, causing its reduced digestibility [7]. This activity of TA results from the great number of hydroxyl groups and is responsible for the inhibition of digestive enzymes, which loose their catalytic activity due to configuration changes [8]. Therefore, TA is traditionally classified as an antinutritional factor for animals, because it negatively affects growth performance and is toxic in high doses. The effects of TA include alterations in morphology and physiology of internal organs such as the liver, intestine, kidneys and spleen [9,10]. Disorders in organ physiology may be reflected in biochemical blood indices. Currently, growing interest in the use of TA as a health-promoting substance is observed [5,11–13], but there are no results concerning its effect on biochemical blood parameters, which should be evaluated, because they clearly indicate the health status of the organism. These indices might be also affected by dietary protein level, as found in the past for plasma total protein, albumin, lipid profile and tissue iron concentration [14–16]. Considering that TA binds to dietary protein, determination of the interactive effect of both factors on physiological parameters seems to be particularly important. Therefore, the present study was performed to investigate the effect of dietary level of TA and crude protein (CP) on the relative weight of internal organs and biochemical blood profile in rats as model animals. It was specifically hypothesised that TA effects could be modified by dietary protein level.

## Materials and methods

### Ethics statement

All procedures were approved by the Third Warsaw Local Ethics Committee for Animal Experimentation (Warsaw University of Life Sciences-SGGW, Warsaw, Poland), according to the principles of the European Union and Polish Law on Animal Protection.

### Rats, diets and experimental design

The experiment was performed on 72 ten-week-old male Crl:W (Han) Wistar rats, divided into 12 groups, each of an average initial body weight of 189 ± 1.4 g. Rats were fed casein-based diets with 10% or 18% of CP and supplemented with 0, 0.25, 0.5, 1, 1.5 or 2% of TA (Table 1). Animals were kept individually in wire-bottom metabolic cages to collect faeces for analysis of protein digestibility, as described previously [7]. Housing temperature was maintained at 22 ± 1°C and the light regimen was 12 h light/12 dark. Animals had free access to feed and water throughout the experiment.

**Table 1:** Composition of experimental diets (%).

CP^1^ level, %	10						18					
TA^2^ level, %	0.0	0.25	0.5	1.0	1.5	2.0	0.0	0.25	0.5	1.0	1.5	2.0
Casein	12.9	12.9	12.9	12.9	12.9	12.9	23.3	23.3	23.3	23.3	23.3	23.3
Tannic acid	0.0	0.25	0.5	1.0	1.5	2.0	0.0	0.25	0.5	1.0	1.5	2.0
Corn starch	53.7	53.5	53.2	52.7	52.2	51.7	43.3	43.1	42.8	42.3	41.8	41.3
Sucrose	12.0	12.0	12.0	12.0	12.0	12.0	12.0	12.0	12.0	12.0	12.0	12.0
Pectin	8.0	8.0	8.0	8.0	8.0	8.0	8.0	8.0	8.0	8.0	8.0	8.0
Cellulose	4.0	4.0	4.0	4.0	4.0	4.0	4.0	4.0	4.0	4.0	4.0	4.0
Soybean oil	4.3	4.3	4.3	4.3	4.3	4.3	4.3	4.3	4.3	4.3	4.3	4.3
DL-methionine	0.09	0.09	0.09	0.09	0.09	0.09	0.09	0.09	0.09	0.09	0.09	0.09
Mineral mix^3^	3.0	3.0	3.0	3.0	3.0	3.0	3.0	3.0	3.0	3.0	3.0	3.0
Vitamin mix^4^	2.0	2.0	2.0	2.0	2.0	2.0	2.0	2.0	2.0	2.0	2.0	2.0

^1^Crude protein

^2^Tannic acid

^3^AIN-93G Mineral Mix (MP Biomedicals, Inc., Eschwege, Germany); %: calcium carbonate 35.7; monopotassium phosphate 19.6; potassium citrate monohydrate 7.078; sodium chloride 7.4; potassium sulphate 4.66; magnesium oxide 2.4; ferric citrate 0.606; zinc carbonate 0.165; manganese carbonate 0.063; copper carbonate 0.03; potassium iodate 0.001; sodium selenate, anhydrous 0.00103; ammonium molybdate·4H_2_O 0.000795; sodium metasilicate·9H_2_O 0.145; chromium potassium sulfate·12H_2_O 0.0275; lithium chloride 0.00174; boric acid 0.008145; sodium fluoride 0.00635; nickel carbonate 0.00318; ammonium vanadate 0.00066; powdered sugar 22.1.

^4^AIN-93-VX Vitamin Mix (MP Biomedicals, Inc., Eschwege, Germany); g/kg: nicotinic acid 3.00; D-calcium pantothenate 1.60; pyridoxine HCl 0.70; thiamine HCl 0.60; riboflavin 0.60; folic acid 0.20; D-biotin 0.02; vitamin B_12_ (0.1% triturated in mannitol) 2.50; α-tocopherol powder (250 IU/g) 30.00; vitamin A palmitate (250 000 IU/g) 1.60; vitamin D_3_ (400 000 IU/g) 0.25; phylloquinone 0.075; powdered sucrose 959.655.

### Sample collection

After 21 days of the experiment, the rats were sacrificed by CO_2_ asphyxiation and cervical dislocation. Blood samples were taken into heparinised tubes by cardiac puncture, centrifuged (3350 g, 10 min) and plasma was stored at -40°C for further analyses. Internal organs (liver, pancreas, stomach, small intestine, caecum, spleen, kidneys) were excised, cleaned and weighed. Digestive organs were emptied by squeezing out the digesta, flushed with physiological saline, blotted dry and weighed.

### Biochemical blood parameters

Blood plasma was analysed for: total protein, albumin, urea, uric acid, creatinine, cholesterol, high-density lipoprotein, triacylglycerols, total bilirubin, alanine aminotransferase, aspartate aminotransferase, γ-glutamyltransferase (γ-GT), alkaline phosphatase (ALP), creatine kinase (CK), amylase, cholinesterase, lactate dehydrogenase, glucose, chloride, phosphorus, iron, calcium and magnesium. All parameters were determined spectrophotometrically on a MAXMAT PL multidisciplinary diagnostic platform (Erba Diagnostics France SARL, Montpellier, France) using ready-to-use reagents (ELITech Group, Puteaux, France).

### Statistical analysis

Data were analysed by two-way analysis of variance followed by Duncan’s multiple range test using the STATGRAPHICS^®^ Centurion XVI ver. 16.1.03 statistical package (StatPoint Technologies, Inc., Warrenton, Virginia, USA). The significance level was set at P < 0.05.

## Results

All TA diets significantly increased (P < 0.05) the relative weight of caecal tissue compared to non-supplemented diets. The greatest differences were found between control animals and rats fed diets with 1.5% and 2% of TA (Table 2, S1 Table). The dietary level of CP significantly affected the relative weight of the stomach, small intestine, caecum, kidneys and spleen (P < 0.05). Rats fed the 10% CP diets had a higher weight of the stomach, small intestine and caecum, but lower of kidneys and spleen, as compared with the animals administered the 18% CP diets. There was no effect of CP level on the relative weight of the liver and pancreas. The relative weight of internal organs was not affected by the interaction.

**Table 2:** Relative weight of internal organs (g/100 g body weight) of rats. Data are presented as mean ± SD.

Experimental factors		Stomach	Small intestine	Caecum	Liver	Pancreas	Kidneys	Spleen
CP^1^ level, %	TA^2^ level, %							
10	0.0	0.41* ± 0.04	2.83* ± 0.54	0.28*^a^ ± 0.04	3.79 ± 0.41	0.34 ± 0.07	0.63* ± 0.03	0.21* ± 0.01
	0.25	0.46* ± 0.04	3.43* ± 0.59	0.34*^b^ ± 0.03	3.96 ± 0.36	0.36 ± 0.06	0.68* ± 0.04	0.19* ± 0.02
	0.5	0.44* ± 0.02	3.14* ± 0.44	0.33*^b^ ± 0.06	4.07 ± 0.40	0.38 ± 0.04	0.65* ± 0.07	0.22* ± 0.04
	1.0	0.45* ± 0.04	3.47* ± 0.52	0.36*^b^ ± 0.03	4.15 ± 0.24	0.42 ± 0.09	0.69* ± 0.05	0.20* ± 0.01
	1.5	0.45* ± 0.05	3.13* ± 0.34	0.34*^b^ ± 0.06	4.16 ± 0.30	0.40 ± 0.12	0.66* ± 0.06	0.19* ± 0.01
	2.0	0.47* ± 0.05	3.42* ± 0.53	0.44*^c^ ± 0.04	3.93 ± 0.25	0.40 ± 0.11	0.67* ± 0.04	0.20* ± 0.03
18	0.0	0.40 ± 0.05	2.77 ± 0.50	0.27^a^ ± 0.02	3.88 ± 0.37	0.38 ± 0.06	0.73 ± 0.05	0.22 ± 0.01
	0.25	0.42 ± 0.03	2.76 ± 0.37	0.30^b^ ± 0.03	4.07 ± 0.37	0.40 ± 0.07	0.70 ± 0.07	0.20 ± 0.04
	0.5	0.42 ± 0.02	3.09 ± 0.49	0.31^b^ ± 0.03	4.00 ± 0.13	0.37 ± 0.05	0.70 ± 0.04	0.22 ± 0.02
	1.0	0.40 ± 0.05	2.84 ± 0.52	0.33^b^ ± 0.04	4.02 ± 0.48	0.37 ± 0.06	0.68 ± 0.03	0.23 ± 0.02
	1.5	0.44 ± 0.04	3.18 ± 0.73	0.36^b^ ± 0.08	4.17 ± 0.46	0.42 ± 0.09	0.70 ± 0.04	0.21 ± 0.05
	2.0	0.43 ± 0.05	2.97 ± 0.62	0.38^c^ ± 0.05	4.24 ± 0.45	0.37 ± 0.06	0.68 ± 0.04	0.22 ± 0.02
SEM		0.005	0.064	0.007	0.042	0.009	0.006	0.003
ANOVA: P values								
CP level		< 0.001	0.018	0.027	0.529	0.862	< 0.001	0.037
TA level		0.088	0.465	<0.001	0.365	0.648	0.945	0.205
Interaction		0.720	0.362	0.421	0.732	0.593	0.080	0.790

^1^Crude protein.

^2^Tannic acid.

*Overall mean for rats fed diets with 10% of CP (n = 36) differs significantly from that of rats fed diets with 18% of CP (n = 36).

^a,b^Lowercase letters refer to the overall means calculated for each level of TA (n = 12), regardless of CP level. Overall means with different letters differ significantly.

TA level significantly affected albumin and phosphorus concentration, and the activity of CK in blood (Table 3, S2 Table). Albumin concentration was higher in rats fed diets with 0.25% and 0.5% of TA than in rats given diets supplemented with 2% of TA (P < 0.05), whereas phosphorus level was higher in rats on the 0.25% and 2% TA diets than in animals on the 1% TA diets (P < 0.05). Feeding diets with 2% of TA significantly reduced the activity of CK compared to rats fed diets without TA and with 0.5%, 1%, and 1.5% of TA (P < 0.05). Rats fed diets containing 10% of CP had a significantly higher activity of ALP, amylase and γ-GT, and the concentration of iron and cholesterol, but a lower level of urea and uric acid in blood plasma, compared to animals administered diets with a higher CP content (P < 0.05). The effect of interaction was only found with respect to cholinesterase activity (P < 0.05). Supplementation of lower protein-diets with 0.25% and 0.5% of TA increased cholinesterase activity compared to the control group and rats given diets with 1%, 1.5%, and 2% of TA, whereas all TA levels reduced cholinesterase activity in rats on higher protein-diets compared to the control group. Other biochemical blood parameters were not significantly affected by the experimental factors (data not shown).

**Table 3:** Biochemical blood parameters of rats^1^. Data are presented as mean ± SD.

Experimental factors		Albumin, g/L	Urea, mmol/L	Uric acid, μmol/L	Cholesterol, mmol/L	ALP^4^, U/L	γ-GT^5^, U/L	Amylase, U/L	CK^6^, U/L	CHE^7^, U/L	Phosphorus, mmol/L	Iron, μmol/L
CP^2^ level, %	TA^3^ level, %											
10	0.0	36^a^^b^ ± 3.2	4.7* ± 1.1	170* ± 123	1.6* ± 0.5	369* ± 100	1.7* ± 1.4	931* ± 223	126^b^ ± 27	238^A^^B^ ± 46	2.49^a^^b^ ± 0.58	29.6* ± 15.5
	0.25	36^b^ ± 0.9	4.6* ± 0.6	171* ± 35	1.6* ± 0.3	428* ± 79	1.3* ± 2.0	794* ± 207	99^a^^b^ ± 39	267^A^^B^ ± 49	2.69^b^ ± 0.13	40.1* ± 13.4
	0.5	37^b^ ± 3.3	5.1* ± 0.9	152* ± 50	1.7* ± 0.3	376* ± 85	0.8* ± 2.0	906* ± 155	138^b^ ± 62	291^B^ ± 36	2.51^a^^b^ ± 0.22	33.4* ± 14.0
	1.0	34^a^^b^ ± 1.4	4.4* ± 0.5	162* ± 44	1.5* ± 0.2	513* ± 259	0.5* ± 0.8	924* ± 132	90^b^ ± 33	242^A^^B^ ± 42	2.16^a^ ± 0.39	43.4* ± 13.8
	1.5	35^a^^b^ ± 0.9	3.7* ± 0.6	150* ± 46	1.6* ± 0.2	418* ± 234	2.4* ± 2.4	922* ± 101	148^b^ ± 96	228^A^^B^ ± 36	2.31^a^^b^ ± 0.47	32.5* ± 17.6
	2.0	33^a^ ± 1.6	3.6* ± 1.6	113* ± 43	1.6* ± 0.3	315* ± 28	1.6* ± 1.3	734* ± 105	47^a^ ± 11	204^A^ ± 58	2.64^b^ ± 0.54	32.4* ± 12.6
18	0.0	35^a^^b^ ± 1.4	6.0 ± 1.7	171 ± 48	1.3 ± 0.2	351 ± 58	0.7 ± 1.1	768 ± 168	126^b^ ± 40	291^B^ ± 50	2.59^a^^b^ ± 0.19	24.9 ± 14.3
	0.25	37^b^ ± 1.5	6.4 ± 1.3	212 ± 99	1.4 ± 0.3	323 ± 73	0.7 ± 0.8	895 ± 93	84^a^^b^ ± 49	224^A^ ± 33	2.76^b^ ± 0.43	18.2 ± 11.2
	0.5	35^b^ ± 1.2	6.6 ± 0.7	230 ± 93	1.2 ± 0.1	319 ± 40	0.9 ± 1.1	754 ± 105	88^b^ ± 64	213^A^ ± 24	2.56^a^^b^ ± 0.29	17.9 ± 4.5
	1.0	36^a^^b^ ± 3.2	6.4 ± 0.9	189 ± 92	1.2 ± 0.3	350 ± 158	0.4 ± 0.8	799 ± 212	132^b^ ± 75	264^A^^B^ ± 77	2.32^a^ ± 0.28	17.2 ± 4.3
	1.5	35^a^^b^ ± 1.7	6.9 ± 1.5	184 ± 62	1.3 ± 0.2	295 ± 64	1.2 ± 0.9	715 ± 253	92^b^ ± 69	217^A^ ± 29	2.51^a^^b^ ± 0.28	17.1 ± 5.8
	2.0	35^a^ ± 1.1	7.0 ± 0.5	169 ± 71	1.2 ± 0.3	313 ± 68	0.5 ± 0.8	780 ± 131	67^a^ ± 19	228^A^^B^ ± 50	2.75^b^ ± 0.40	20.2 ± 6.9
SEM		0.25	0.18	8.6	0.04	15.4	0.17	20.2	6.8	6.1	0.046	1.67
ANOVA: P values												
CP level		0.854	< 0.001	0.026	< 0.001	0.011	0.048	0.037	0.433	0.611	0.212	< 0.001
TA level		0.033	0.849	0.554	0.876	0.346	0.325	0.691	0.032	0.112	0.027	0.863
Interaction		0.109	0.091	0.857	0.905	0.587	0.798	0.145	0.199	0.015	0.996	0.338

^1^Only parameters that were significantly affected by the experimental factors are presented.

^2^Crude protein.

^3^Tannic acid.

^4^Alkaline phosphatase.

^5^γ-glutamyltransferase.

^6^Creatine kinase.

^7^Cholinesterase.

*Overall mean for rats fed diets with 10% of CP (n = 36) differs significantly from that of rats fed diets with 18% of CP (n = 36).

^a,b^Lowercase letters refer to the overall means calculated for each level of TA (n = 12), regardless of CP level. Overall means with different letters differ significantly.

^A,B^Cholinesterase activity was affected only by the interaction; therefore, superscripts refer to the group average (n = 6) and means within column with different letters differ significantly.

## Discussion

The effect of diet on the relative weight of internal organs reflects changes in their metabolic activity and structure caused by protein, fibre, antinutritional factors and toxins [17]. Owing to the chemical structure, TA cannot be directly absorbed in the intestine but it is hydrolysed in the digestive tract by intestinal or bacterial enzymes. As a polyphenolic compound, TA is conjugated in the intestine during absorption and then in the liver, which facilitates its removal with bile and urine. However, polyphenols, including TA, have the ability to penetrate tissues, especially those involved in their metabolism, i.e. the intestine and the liver [18]. As reported previously [7], TA had no effect on feed intake, but reduced body weight gain of rats and worsened feed conversion ratio. However, the lack of effect of TA level on liver, pancreas and kidney weights may indicate that dietary supplementation with 0.25% to 2% of TA does not generate any toxic effects. The caecum was the only organ, which relative weight was affected by TA supplementation. Previously, it was demonstrated that TA increased the relative weight of caecal digesta and short-chain fatty acid production [7]. Therefore, enlargement of caecal tissue may be explained by reduced digestibility of nutrients and their accumulation in the caecum. This, in turn, intensified microbial activity and increased concentration of these acids that could stimulate epithelial cell proliferation. Similar effects on caecal tissue were also reported in regards to other nutritional factors modulating fermentative processes [19–21].

Greater relative weights of the stomach, small intestine and caecum in rats fed diets with 10% of CP may be related to worse protein utilisation for muscle growth. This would be consistent with previously shown smaller body weight gains of rats fed diets with lower CP content [7]. This effect may result from a greater need to maintain viscera metabolism than to increase muscle weight. In rats, 43% of protein synthesised in the organism, originates from the liver and digestive tract [22]. However, in the present study, the relative weight of the liver and pancreas was not affected by CP level, which might be related to the age of rats at the beginning of the trial and duration of the experiment. In younger animals, feeding diets with higher protein content increased liver weight [23], but the opposite effect of such diets was also observed [24], indicating that the liver response to dietary protein is not uniform.

The present study demonstrated a hypertrophic effect of higher CP level on kidneys, which could be explained by the metabolism of larger quantity of nitrogenous compounds in these organs. This is consistent with earlier studies [19,25] and corresponds to higher blood urea and uric acid concentrations in rats fed with the 18% CP diets. Feeding higher-protein diets also increased the relative spleen weight, which might indicate the immune system response [17].

The biochemical blood profile was only slightly affected by TA level. Feeding diets with 2% of TA considerably reduced the activity of CK, a cardiac marker enzyme, which was in agreement with previous findings of cardioprotective effects of TA and other polyphenols [4,26,27]. It has recently been suggested that TA maintains the integrity of the cardiac cell membrane and prevents the leakage of CK and other cytosolic enzymes into the bloodstream [4], which seems to be confirmed by the results of our study. TA may exert its cardioprotective effect by reduction of Bax/Bcl-2 ratio, expression of c-fos and c-jun, inhibition of NF-κB activation, as well as due to antioxidative and antiinflammatory properties [4]. Cellular mechanism of TA activity also include inhibition of L-type Ca^2+^ channels in cardiomyocytes [27]. The effect of TA on blood phosphorus level is difficult to explain and it is not clear why feeding diets with 0.25% and 2% of TA elevated its concentration compared to rats on the 1% TA diets. Analyses of bone metabolism or parathyroid gland activity could provide a better insight into this phenomenon.

Despite the lack of influence on the liver weight, diets with 2% of TA slightly reduced the concentration of albumin, which is synthesised predominantly in this organ. This effect corresponds with earlier findings of reduced protein digestibility [7], enhancement of catabolic processes [1] and impairment of protein synthesis [10] in the liver of animals fed diets containing tannins. It remains to be elucidated why alterations in albumin concentration, observed in the present study, do not cause any changes in the total protein content in blood plasma.

Dietary supplementation with TA had no effect on transaminase activities, which was in line with unaffected relative liver weight and indicated that hepatocyte damage did not occur. However, the activity of cholinesterase, synthesised in the liver [28], was altered by the interaction between TA and CP levels. Cholinesterase activity may be influenced by many factors, including protein-energy malnutrition, stress, hepatic injury or inflammatory state [28], but its inhibition may be only evidence of absorption TA metabolite absorption into the bloodstream, as it is for organophosphorus or carbamate esters [29]. Thus, the interactive effect, shown in the present study, may suggest that feeding higher-protein diets to rats facilitates absorption of TA metabolites, even at low dietary TA levels.

In the present study, it was shown that dietary protein level was an important factor shaping biochemical blood profile, as it affected 7 out of 23 analysed parameters. Contrary to the aforementioned urea and uric acid, feeding diets with 18% of CP reduced cholesterol, iron, amylase, γ-GT and ALP levels. Lower cholesterol concentrations in animals fed diets with a higher protein content were observed in the past [14], and this phenomenon was confirmed in our study. This effect probably results from a greater bacterial production of propionic acid in the caecum of rats, which was described previously [7]. It is known that propionic acid inhibits liver synthesis of cholesterol from acetic acid, probably via the inhibition of 3-hydroxy-3-methylglutaryl-CoA reductase, which is a rate-limiting enzyme in cholesterol synthesis [30,31].

The effect of CP level on iron concentration and enzyme activities in blood plasma is poorly understood. Higher ALP levels were observed during intensive body growth due to bone formation [32], but in our experiment, it was rather related to the greater relative weight of the small intestine of rats fed the 10% CP diets, as the intestinal ALP isoenzyme was shown to contribute to the total ALP activity in blood plasma [33].

Considering the kidney’s role in clearing the bloodstream of amylase [34], it could be expected that smaller kidneys in rats fed the 10% CP diets were less efficient than those in rats administered the 18% CP diets. This could be the reason of the elevated activity of this enzyme and could contribute to higher levels of iron and γ-GT. On the other hand, dietary protein level did not affect creatinine concentration, which is used as a marker of glomerular function [35]; thus the effect on the above-mentioned indices remains to be elucidated.

## Conclusions

The findings of the present study suggested that basic physiological parameters, i.e. the relative weight of internal organs and biochemical blood profile of rats were not affected by the interaction between TA and CP levels, with the exception of cholinesterase activity. TA, up to the 2% dietary level, induced caecal hypertrophy and could act as a cardioprotective agent, as demonstrated by decreased CK activity. CP level had a greater impact on rat physiology than TA because it affected not only the caecum, but also the stomach, small intestine, kidneys and spleen. Feeding diets with a higher CP content had a hypocholesterolemic effect and additionally influenced those biochemical blood indices that might be associated with kidney physiology. However, this phenomenon is unclear and requires further investigations.

## Supporting information

S1 Table Raw and relative weight of organs. Raw data.
(XLS)

S2 Table Biochemical blood indices. Raw data.
(XLS)
